# The Form of Morphemes: MEG Evidence From Masked Priming of Two Hebrew Templates

**DOI:** 10.3389/fpsyg.2018.02163

**Published:** 2018-11-12

**Authors:** Itamar Kastner, Liina Pylkkänen, Alec Marantz

**Affiliations:** ^1^Department of English and American Studies, Faculty of Language, Literature and Humanities, Humboldt-Universität zu Berlin, Berlin, Germany; ^2^Department of Linguistics, New York University, New York, NY, United States; ^3^Department of Psychology, New York University, New York, NY, United States; ^4^NYUAD Institute, New York University Abu Dhabi, Abu Dhabi, United Arab Emirates

**Keywords:** Hebrew, lexical access, masked priming, MEG, root and pattern morphology

## Abstract

Studies of lexical access have benefited from comparisons between languages like English, which shows concatenative morphology, and Semitic languages showing non-concatenative morphology of roots and patterns. Morphological decomposition in Semitic has previously been probed using masked priming, originally developed to investigate concatenative morphology. However, studies conducted on Semitic languages have often targeted Semitic-specific questions, such as whether the root and the verbal template prime lexical access. The overall consequence of these studies for our understanding of lexical access remains unclear. In two experiments on Hebrew using MEG, we demonstrate that a verbal form which is orthographically and phonologically indistinguishable from non-verbal forms is primed by other verbs in the same template but not by similar nouns and adjectives. These results suggest that masked priming taps into more than just visual forms but reflects morphological content, even if this content is abstract, showing no distinct orthographic or phonological marking.

## Introduction

Theories of morphology often assume that a word is made up of a sequence of morphemes concatenated into a string. Studies of lexical access have capitalized on this intuition, examining how the different parts of a word are taken apart, looked up in the mental lexicon, and recombined. In such studies, a crucial distinction is made between the lexical stem on the one hand and affixes that serve to derive related words on the other. By keeping the stem constant and modifying the affixes, or by keeping the affixes constant and modifying the stem, it is possible to better understand how derivational processes progress. For example, the nominalizing suffix -*er* in *writer* and the arbitrary string (“pseudo-suffix”) -*er* in *brother* are processed similarly as primes in masked priming, suggesting that obligatory morpho-orthographic decomposition into stems and affixes happens early on during processing, before the meaning of the postulated morphemes is consulted (Rastle et al., 2004; Lewis et al., 2011).

Yet not all languages fit this sequential mold, at least not on the face of things. Semitic languages such as Hebrew exhibit non-concatenative morphology: the consonants and vowels of the word spell out distinct morphemes and are interleaved in systematic ways. For example, the consonants G-D-L appear in the verb *gadal* “grew,” the related verbs *gidel* “raised” and *higdil* “enlarged,” the related nouns *godel* “size” and *migdal* “tower,” and the related adjective *gadol* “large.” In traditional descriptions, G-D-L is called the consonantal root. The patterns of prefixes and vowels that derive words from this root are constrained to different degrees: inserting the vowels -*i-e*- between the root consonants often results in a transitive verb (as in *gidel* “raised”), but that is not the case for each and every root (contrast *kipets* “jumped around,” which is intransitive). Similarly, an abstract noun can often be formed by inserting the vowels -*o-e*- (as in *godel* “size”), but this too varies from root to root. Many words can be derived using the same “root,” and their meaning need not be immediately transparent: one nominalizing pattern results in *gdila* “growth,” while the other results in *gidul* “tumor.” Importantly, for all Semitic languages, while there are potentially many nominalizing patterns, the number of verbal patterns is restricted. In Modern Hebrew there are seven such verbal *templates*.

This morphological system will be illustrated focusing on two of the seven templates in Modern Hebrew, *hiXYiZ* and *XaYaZ*. These names allude to the positions of the root consonants (*X*, *Y*, and *Z*), the template-specific vowels (e.g., -*a,a-* in *XaYaZ*) and template-specific prefixes (e.g., *hi-* in *hiXYiZ*). Even though this information is available in the phonology, it is not all discernible from the orthography. For example, the template *hiXYiZ* is written with the consonantal *h* and the second *i*, but without the first *i*: HXYIZ. The verb *higdil* “enlarged” in this template is written HGDIL. This template may be classified as “overt” since the orthography makes it clear that the written word should be read as a verb in *hiXYiZ*. The second of the two templates, *XaYaZ*, can be exemplified using the verb *gadal* “grew” in the same root, G-D-L. This template has no overt orthographic clues to the morphology: the word is written GDL, in effect showing nothing but the three root consonants. Even though GDL is a verb, there is nothing explicitly verbal about the orthographic form. Having only three consonants as its written form is not enough to single out a given word as a verb, since the same orthographic form is utilized by other lexical categories, namely nouns (e.g., BSR, pronounced *basar*, “meat”) and adjectives (e.g., KTN, pronounced *katan*, “small”). We will reserve the term “template” to refer to the verbal patterns, of which we focus on these two, *XaYaZ* and *hiXYiZ*. The template *XaYaZ* may be seen as “covert,” since there is no orthographic information classifying the word as a verb in this template. The question then arises whether speakers are sensitive to the distinction between verbs and other categories early in visual word recognition, even when this distinction is not signaled by the orthography or the phonology.

With these distinctions to be found between similar orthographic forms, Semitic languages have the potential to contribute much to our understanding of lexical processing. Presumably, any model of the mental lexicon that is appropriate for the concatenative morphology of a language such as English should be applicable to the non-concatenative morphology of a language such as Hebrew; if not, it is necessary to explain how and why the cognitive processes might be different for the two languages. Specifically, assume that the mental lexicon is arranged in whatever way allows it to distinguish stems from affixes as in English. This means that the different kinds of morphemes are listed in one way or another. In reading, the word is decomposed into stem and affix based on the reader’s knowledge of the affixal forms in her language: word-final -*er* is immediately parsed as a suffix, whether in *worker* or *brother*, although this initial judgment can later be overturned (Rastle et al., 2004; Fruchter and Marantz, 2015). What would this division look like for Hebrew? One tempting answer would be that roots are listed as consonantal tuples, as with G-D-L, similarly to stems. Affixes would then also be listed. For example, the -*a-a*- combination in *gadal* “grew” would have to be listed as one kind of verbalizing affix, -*i-e*- in *gidel* “raised” would be listed as another, *hi-i* in *higdil* “enlarged” would be another, and so on.

But this initial characterization cannot be the whole story, because the vowels do not map uniformly to functions. While *-a-a-* is a verbalizer in *gadal* “grew,” the same vowel combination serves to derive adjectives and nouns with other roots. This phenomenon is familiar from other languages: for example, in English it is necessary to distinguish between the agentive suffix -*er* in *writer* and the comparative suffix -*er* in *bigger* if we are to distinguish their functions. The mapping from form to function is often not deterministic, requiring more explicit theories of what kind of relationship is embodied between the orthographic representation and the function of the morpheme. In Semitic this question is brought out more sharply when the affix is not even orthographically visible, as with forms like GDL (*gadal*, “grew”), KTN (*katan*, “small”) and BSR (*basar*, “meat”). What this means is that the affixes in “grew,” “small” and “meat” are represented similarly in the orthography: not at all.

The Semitic system presents the following test case for theories of morphological processing. Upon encountering a written word, the reader must realize how it is pronounced, sometimes without overt cues. But concurrently, she must also map this pronunciation to the correct pattern, which contains morphosyntactic and semantic information. We report here on two masked priming experiments that used magnetoencephalography to investigate what the makeup of the mental lexicon is like based on the morphological processing of a non-concatenative language, testing whether lexical access differs between “root-and-template” languages (such as Hebrew) and “stem-and-affix” languages (such as English). Our study concentrates on the difference between a verb like *gadal* “grew,” with the orthographic representation consisting of three consonants (GDL), and a noun like *basar* “meat,” with a similar orthographic representation (BSR). Whatever morphological structure distinguishes these two words is not available from the phonology or the orthography. It follows that if priming is sensitive to the visual form of affixes, there should be no difference between the two words. However, if priming is sensitive to an abstract affix which has a null form, such as the verbalizer in “grew,” template priming will obtain even without overt formal cues to the template or grammatical category.

## Lexical Decomposition

Studies of lexical access have provided support for a “full decomposition” model of reading in which a word is decomposed into discrete constituent morphemes (Taft and Forster, 1975) and then recombined (Taft, 1979, and more recently Fruchter and Marantz, 2015; Neophytou et al., 2018). Not all models of lexical access subscribe to a decompositional view. Connectionist models and their contemporary incarnations have offered various ways of linking form and meaning without decomposition into constituents (Seidenberg and McClelland, 1989; Plaut et al., 1996; Baayen et al., 2011; Marelli et al., 2015; Amenta et al., 2017). We will not enter this debate directly, but we do note that even a-morphous models have been critiqued as encoding morpheme-level information (Marantz, 2013), meaning these models still make reference to symbolic representations. In addition, it has recently been argued that such models are unable to account for certain morphological family effects in Hebrew which rely on the notion of a root (Deutsch and Kuperman, 2018). A growing body of work now supports the early decomposition hypothesis, according to which the visual form is parsed into constituents which are then looked up. This conclusion is based on evidence from masked priming in behavioral studies (Rastle et al., 2004; Rastle and Davis, 2008; Crepaldi et al., 2010, 2013, 2016) and ERP studies (Lavric et al., 2007; Royle et al., 2012; Morris et al., 2013; Beyersmann et al., 2014), all of which exhibit early morphological priming effects.

Using MEG, an early correlate of lexical decomposition has been identified, namely the M170 (Zweig and Pylkkänen, 2009). This component is modulated by the transition probability from stem to affix roughly 170 ms post target onset, for affixed words like *farmer* and *classic* (Solomyak and Marantz, 2010; Fruchter and Marantz, 2015) as well as for “pseudo-affixed” words like *corner* and *panic* (Lewis et al., 2011; Whiting et al., 2014). Fruchter et al. (2013) obtained masked priming effects in the M170 for both regular and irregular past tense verbs, indicating that the effect generalizes to irregular inflection. Spatially, this effect localizes to the left fusiform gyrus and overlaps to a large degree with responses associated with the Visual Word Form Area (Dehaene et al., 2002; Gwilliams et al., 2016). This is the first stage of processing morphologically complex words as being morphologically complex.

Following the decomposition stage, the parsed morphemes are looked up. A possible neural component of lexical access has been identified and termed the M350. This component is modulated by base frequency of the stimulus (Embick et al., 2001) and has been localized to the middle and superior left temporal regions (Solomyak and Marantz, 2010; Lewis et al., 2011; Fruchter et al., 2013; Fruchter and Marantz, 2015). It is at this point that semantic priming effects have also been found, localized to the left middle temporal gyrus in a number of MEG studies (Friederici, 2002; Whiting et al., 2014). This region has also shown increased activation in semantic judgment (Binder et al., 1997) and priming tasks (Devlin et al., 2004; Gold and Rastle, 2007) in a number of fMRI studies.

This two-stage model of lexical access leads us to focus on two proposed neural correlates of lexical processing in the left hemisphere: activation correlated with the transition probability from stem to affix in the fusiform gyrus (M170) and activation correlated with lexical lookup in a mid-temporal region (M350). Laszlo and Federmeier (2014) have found a number of effects reflecting lexical access in an ERP study, in line with the model described here: form features appeared earlier (as late as 180–210 ms) and semantic effects later (300–340 ms). Under this model, form-based decomposition happens about 170 ms post stimulus onset and is associated with activation in the fusiform gyrus (Visual Word Form Area). Lexical access then occurs around 350 ms post stimulus onset, associated with activation in a medial part of the left temporal lobe.

### Previous Results for Roots and Templates

As alluded to above, studies of lexical access in Semitic languages must manipulate different factors than stems and affixes, since the Semitic morphological system is based on different morphophonological premises. On a view in which a written word is decomposed into the visual forms of morphemes, the relevant questions are whether roots and templates are themselves morphemes, with the former corresponding to stems and the latter to affixes. The evidence supports this view for roots, whereas the status of templates has been less clear.

In a number of masked priming experiments in Hebrew employing lexical decision, Frost and colleagues found evidence for root priming: nouns primed other nouns sharing the same root (Frost et al., 1997) and verbs primed other verbs sharing the same root (Deutsch et al., 1998), regardless of semantic similarity (see Deutsch, 2016, for similar results from a picture naming study). Pattern priming was limited to verbal templates: verbs primed other verbs in the same template (Deutsch et al., 1998) but nouns did not prime other nouns in the same pattern (Frost et al., 1997). Deutsch et al. (1998) additionally found that a pseudo-word verb does prime the same template with another root (the non-existent *hgmir* primes HLBIŠ “dressed”) when compared to a control condition in which the prime differed from the target by one root consonant. The exact results depend on the consonants; a two-consonant representation of the root leads to priming in some cases but not others (Frost et al., 2000a; Velan et al., 2005).

Additional studies have supported the claim that this priming effect is morphological in nature rather than purely formal, i.e., not based on orthographic similarity, as indicated by findings from lexical decision and sentence production tasks (Frost et al., 2005; Velan and Frost, 2011). Root priming in Hebrew has so far only been found in “overt” templates, those distinguished by affixes. No findings have been reported for priming of “covert” templates like XYZ. Eye tracking studies have led to comparable results, privileging the root and overt verbal templates (Deutsch et al., 2000, 2003); facilitation effects had not been found for overt nominal patterns (Deutsch et al., 2005), although recent findings indicate that nominal patterns might play a similar role in lexical access after all (Deutsch and Malinovitch, 2016; Deutsch et al., 2016). The privileged status of the root in Hebrew was also emphasized by experiments utilizing cross-modal priming (Frost et al., 2000b) and picture-word interference tasks (Deutsch and Meir, 2011; Kolan et al., 2011; Deutsch, 2016). Sensitivity to shared roots was found in a pair of fMRI studies as well, localizing BOLD activation to several regions in the left hemisphere in lexical relatedness judgments (Bick et al., 2008) and masked priming (Bick et al., 2010), while a behavioral study showed slower reaction times (RTs) to pseudo-words derived from existing roots than nonce roots in lexical decision, again in line with the status of the root as a morpheme (Yablonski and Ben-Shachar, 2016).

Turning to evidence for the status of roots and templates in related languages, behavioral studies of Modern Standard Arabic have likewise found priming effects for roots and verbal templates that did not hold for nominal patterns or other controls (Boudelaa and Marslen-Wilson, 2005, 2011, 2015), although orthographic similarity effects have been noted as well (Perea et al., 2015). Evidence from aphasia has similarly implicated the root as a basic element of morphological processing in Arabic (Prunet et al., 2000; Idrissi et al., 2008). An auditory ERP study of Arabic obtained mismatch negativity effects for the root at 160 ms and for the nominal pattern at 250 ms post-divergence, distinguishing the two (Boudelaa et al., 2010). Using MEG in an auditory study, Gwilliams and Marantz (2015) found that Arabic speakers are sensitive to transition probabilities between different consonants in the root in auditory presentation, indicating that speakers are sensitive to Semitic roots as elements with an internal structure made up of individual consonants. As with the Hebrew studies, the emerging consensus is that consonantal roots can be primed and are presumably represented in the mental lexicon of speakers. Results are less clear-cut for templates and depend in large part on the language and experimental design.

The main findings of experimental work on Semitic can be summarized as follows. The consonantal root functions as a typical morpheme for speakers of Semitic languages: it can be primed and it exists on its own level of representation. The status of the verbal template is less clear, however. On the one hand, the template can be primed: a verb in an “overtly” affixed template can be primed by a verb instantiating a different root in the same template. On the other hand, the priming behavior is not robust. Not every template can be primed in every Semitic language tested, and even when the template is primed, the exact interaction with the root seems to influence the result. For instance, pseudo-words prime real words in the same template, but this template was overt, i.e., contained a prefix H- and an infix -I-, in the study of Deutsch et al. (1998). One possible interpretation is that the prefix is primed. Such an explanation would account for this result, but it would not predict other findings from auditory priming in Moroccan and Maltese (Schluter, 2013; Ussishkin et al., 2015) or make any predictions regarding “covert,” non-affixed templates.

### Previous Masked Priming of Stems and Affixes

In masked priming, no lexical-conceptual meaning is believed to be accessed when seeing the prime: semantic priming effects do not generally obtain (though for challenges to this claim see, e.g., Feldman et al., 2009, 2012). Presumably this is because the prime is not looked up in full, voiding its ability to prime a semantically related target. Nevertheless, masked priming does show other priming effects, most notably identity priming. On some level of representation, all masked priming can be thought of as identity priming: the visual form is recognized, making it easier to recognize once the form appears as part of the target.

In English, stem priming has been reported in a number of studies: a stimulus consisting of a stem and one suffix primes a target consisting of the same stem and a different suffix (Rastle et al., 2000, 2004). This result has also emerged from a number of studies in which an English irregular past tense verb primed the infinitival form, e.g., *fell* primed FALL (Crepaldi et al., 2010). Stem priming was also found when the same stem was part of different compounds (Crepaldi et al., 2013; Fiorentino et al., 2015). Perea et al. (2015) obtained evidence that priming ignores the uppercase/lowercase distinction, meaning that pure formal overlap does not drive masked priming alone.

Masked priming has been reported to hold for affixes as well as stems. Controlling for orthographic overlap and priming effects, prefixes (English: Chateau et al., 2002) and suffixes (Spanish: Duñabeitia et al., 2008) both showed faster RTs when preceded by masked primes sharing the affix. For instance, *unable* primed UNFAIR, but no effect was found for the control condition *element*-ELEVATOR.

The model resulting from these findings relates masked priming to form matching (see the synthesis in Amenta and Crepaldi, 2012; Acha and Carreiras, 2014). The past tense can be conveyed by the suffix –*ed* (*worked*) or by an irregular verb (*gave*). In either case, the affix is “stripped” off the stem and the stimulus is decomposed into the form of the stem and the form of the affix. Each of these might then be primed, as indicated by the results mentioned above. Semantic information is not accessed during masked priming, indicating that this priming is the result of formal lookup.

It has been argued that at least some findings in the masked priming literature should be better understood as task effects: the presumed decomposition *corn-er* was supported by shorter response times in a masked priming lexical decision task when compared to an orthographic control such as *broth-el* (Rastle et al., 2004), but not in a masked priming eye tracking study (Marelli et al., 2013). This contrast led Marelli et al. (2013) to suggest that decomposition in masked priming is task-specific, reflecting comprehension plus decision rather than just comprehension. Nevertheless, neurophysiological studies have also employed priming paradigms with lexical decision and found comparable results. In these studies, responses to lexical decision were not part of the dependent variable as such.

In the behavioral literature, facilitation of response times reflects a lower degree of lexical processing for the target having seen a related prime, when compared to that required after an unrelated prime. A similar assumption has been made in recent MEG studies. Matching a formal stimulus in the prime triggers lexical activation; an overt priming study using MEG found that *taught* primes TEACH, leading to reduced activation at the M350, the possible neural correlate of lexical access mentioned above (Stockall and Marantz, 2006). This result is consistent with the hypothesis that complex forms like *taught* are decomposed into the affix and the stem, *teach*, which itself can be primed. Importantly, similar results obtained in a masked priming experiment (Fruchter et al., 2013). Under the model explored here, lookup of orthographic forms happens first, as indexed by the M170, followed by lexical access to the decomposed elements, as indexed by the M350 (see Fruchter and Marantz, 2015, for a finer-grained model). Priming can be seen as facilitating search in the mental lexicon: if the prefix *un*- is seen in the prime, it is activated based on its form, and so *unable* primes UNFAIR, regardless of whether the dependent variable in a given study is behavioral or neurophysiological.

The English results leave open the question of what level of representation morphological priming operates on. Specifically, what are the limits of activation based on the form of affixes: is affix priming the priming of a certain orthographic pattern? Of a phonological pattern, transcribed orthographically? Of an abstract morpheme, or of the features of a morpheme?

The effects discussed above are importantly sensitive to a number of continuous factors, including transition probability from stem to affix (M170), morphological family size and stem frequency (M350). Analyses exploiting these measures therefore rely on appropriate corpora from which these measures can be derived. In Hebrew, to which we turn now, extracting these measures is less immediate due to the complex morphological system and the large degree of homography discussed in the Introduction. While this task is partly tractable for Hebrew nouns (Moscoso del Prado Martín et al., 2005; Deutsch and Kuperman, 2018), carrying it out for verbs would have required an appropriate corpus, parsing software and additional theoretical choices regarding what counts as an affix. As a first step in this direction, then, we opted to perform a masked priming experiment, accepting that some of the gradient characteristics of the neural responses would be lost when using a binary predictor.

Three hypotheses were evaluated in the present study. First, masked priming could be about stored word forms. On this view, *worked* primes WORK because both share the string *work*. Likewise, *taught* primes TEACH because *taught* is decomposed into *teach* and the past tense suffix. For Hebrew, this would mean that the “covert” verbal template XYZ would not show priming at either the M170 or M350, since there is no overt affix whose orthographic form is activated.

On the second hypothesis, priming is about an extra level of abstraction: priming the abstract orthographic form of a lexical element, i.e., the morpheme itself, not just its overt representation. For Hebrew, this would mean that the “covert” template will show priming when compared to a formally identical non-verb. The logic is as follows. When the reader sees a verbal stimulus, she begins to project morphological structure. If the string is unambiguous—if it can only be read as a verb—then it is decomposed into a root CCC_1_ plus a null verbal affix, the abstract form “v_CCC_.” The Hebrew script does not always reflect the morphological makeup of a word unambiguously; in our model of the lexicon, verbs are formed by combining a root with a verbal affix which might be null or overt. If the target is likewise unambiguously a verb, it is parsed into its root CCC_2_ plus the null verbal affix v_CCC_. Under this hypothesis, the null form of the affix is recognized, correlated with activation at the M170. This abstract form next activates the lexical entry for v_CCC_, leading to priming at the M350. For Experiment 2, where a “covert” template is primed, this kind of template priming would mean that masked verbs prime target verbs while masked nouns and adjectives do not prime target verbs. This kind of result would provide theoretical grounding for a category effect, in which verbs prime verbs but not other lexical categories, since the affix carries categorial information (a verbalizer, in this case).

In both cases, the “overt” template is predicted to show priming effects at the M350—assuming it is recognized and undergoes lexical access—and perhaps at the M170 as well, if the formal characteristics of the template can be identified from characters that are not linearly adjacent. This prediction, a partial replication of previous behavioral and EEG work, was tested in Experiment 1. A discrepancy between the findings at the M170 and the M350 might indicate that our understanding of them based on English studies would need to be revised.

A third and final alternative is that priming could be about the abstract features that make up the morpheme. Verbs can be transitive (e.g., *destroy*) or intransitive (e.g., *dance*). If priming is sensitive to the grammatical features of a verb, then a transitive verb would prime an otherwise unrelated transitive verb but not an unrelated intransitive verb. This hypothesis necessitates a theory of what features make up the experimental items and is returned to in Experiment 2.

All three hypotheses aim to account for what kinds of structures are recognized during lexical access. With the status of the root fairly well established for Semitic, the question of how templates are represented remains unanswered, and with it the question of what parts of a word get primed. In order to probe this question more fully, we replicated the behavioral masked priming results for an overt affixed template in Experiment 1, this time using MEG, and investigated the three hypotheses in more detail in Experiment 2.

## Experiment 1

The first experiment tested whether priming effects obtain for the root and template in *hiXYiZ* (orthographically HXYIZ). This is a test of whether an overt, prefixed template in Hebrew shows similar behavior to that of an affix in a language like English.

### Methods

#### Participants

21 right-handed native speakers of Hebrew participated in the study (11 female, mean age 30.9), all with normal or corrected-to-normal vision. All participants had grown up speaking Hebrew in Israel and used Hebrew in their daily lives even when living abroad. All provided written informed consent to participate in the study and were paid for their time.

#### Materials

**FIGURE 1:**
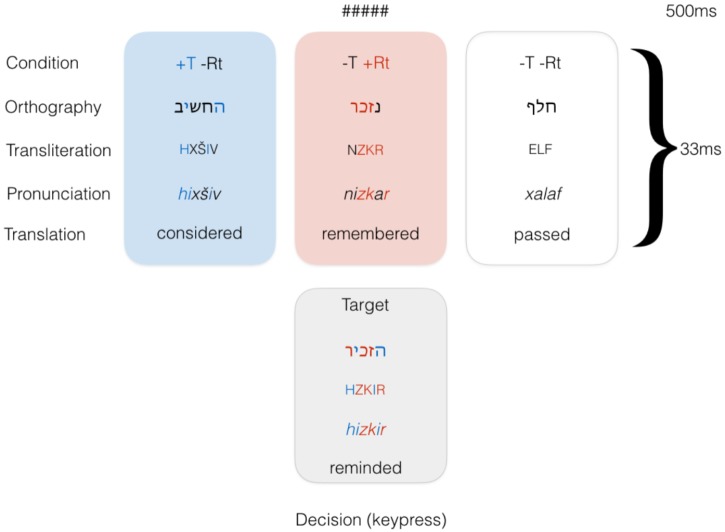
Conditions in Experiment 1.

In our priming design, the target remained constant across conditions and was preceded by three types of primes. Target words were in the HXYIZ template (pronounced *hiXYiZ*), with primes either matching the template in the Shared Template condition (+T-Rt), matching the root in the Shared Root condition (-T+Rt), or being unrelated verb controls in a different template (-T-AS). See Figure 1 for illustration of the conditions and trial structure. Verbs in this template are either transitive or—much less frequently—intransitive verbs indicating a change of state (e.g., “grow pale”). Only transitive verbs were used. No identity condition was introduced; this decision was made in order to prevent the target item from being seen too many times throughout the experiment.

All items were common words used in modern-day Hebrew, as judged impressionistically by the first author and confirmed using corpus and search engine lookups. Surface frequencies were obtained using a 165M-word corpus of Hebrew blogs (Linzen, 2009), to ensure that the data reflected contemporary usage as closely as possible. For additional considerations regarding prime frequencies in Hebrew priming experiments see Frost et al. (2005). Lexical statistics are given in Table 1. None of the differences in frequency between conditions approached significance, with the exception of the pairwise comparison between +T-Rt and -T-Rt, *t*(53.7) = 1.76, *p* < 0.1. Items in the +T-Rt condition showed higher orthographic overlap with the targets (*M* = 2.5) than did items in the -T+Rt condition (*M* = 3.4), *t*(81.9) = 6.27, *p* < 0.001. This difference arises due to the fact that the template shares two consonants across conditions (prefix H- and infix -I-), while the root shares all three root consonants.

**Table 1:** Lexical statistics for stimuli in Experiment 1.

Condition	Word length	Surface frequency per million
+T -Rt	5.0	2.45
-T +Rt	4.2	6.31
-T -Rt	3.8	9.17
Target	5.0	4.60


**Table 2:** Behavioral results for Experiment 1.

Condition	Words		Non-words	
		
	Mean RT	*SD*	Mean RT	*SD*
+T -Rt	652.5	150.1	720.3	162.2
-T +Rt	642.0	154.3	725.3	157.8
-T -Rt	652.9	147.9	719.3	162.4


Stimuli were presented in “unpointed” or “vowelless” script in order to allow for as natural a reading experience as possible. All verbs were in the third person masculine singular past tense, the standard citation form. All items were chosen such that they would be read unambiguously; written Hebrew does not mark vowels, leading to a large number of homographs (for instance, the orthographic form HPNIM could either be read has the verb *hifnim* “internalized” or as the definite noun *ha-panim*, “the face,” so this string was not used; the initial H- and medial -I- do not deterministically indicate a verbal form). Care was taken to select strings that could only be read as the verb in question. This selection criterion narrowed down the available number of verbs considerably, but ensured that stimuli would be perceived as naturally as possible since pointed script is not used by adult speakers of Hebrew. This self-imposed limitation also meant that matching for frequency was not the first concern, though the conditions did not differ from each other in this respect. In any case, since the same target was matched with different masked primes, any imbalance was not expected to affect the results (Frost et al., 2005).

There were 42 word targets and 42 non-word targets, each matched with three possible primes, for a total of 252 items. Non-words were phonologically legal sequences of letters in Hebrew. The order of items was pseudorandomized across participants and only word trials were included in the analysis. See the Supplementary Materials for the materials.

#### Procedure

Subjects lay in a dimly lit, magnetically shielded room and performed a lexical decision task. The subjects saw a string of hash marks (the forward mask, “#####”) for 500 ms followed by the prime which appeared for 33 ms, followed by the target. Stimuli were presented using DMDX (Forster and Forster, 2003). Participants were instructed to respond to the target stimulus as quickly and accurately as possible by pressing one button if it they recognized the string as a word in Hebrew, and another if they thought the string was not a valid word in the language. Primes were displayed in 11-point Arial font and targets in 20-point Arial. Hebrew orthography does not employ an uppercase-lowercase distinction, so different font sizes were used instead (Frost et al., 2005).

The MEG data were recorded using a 157-channel axial gradiometer whole-head MEG system (Kanazawa Institute of Technology, Kanazawa, Japan) at a sampling frequency of 1,000 Hz. The data were filtered between DC and 500 Hz, with a notch filter of 60 Hz. Subjects’ heads were digitized prior to entering the MEG room using a Polhemus Fastrak 3D digitizer (Polhemus, VT, United States). Head positions during the experiment were determined via coils attached to anatomical landmarks.

Experiments 1 and 2 were run concurrently to minimize recording time and make the most use of participants’ time, with items from one experiment serving as fillers for the other; target items were therefore separated from one another by targets in the other experiment, including words and non-words. Recording lasted approximately 25 min.

#### Analysis

##### Behavioral data

Participants’ responses were analyzed for accuracy and RT. Subjects whose mean RT was more than two SDs above the mean RT for all subjects were excluded from the behavioral analysis; this criterion resulted in the removal of one subject. Trials with an RT that was either less than 200 ms, greater than 1,500 ms, or greater than two SDs away from the mean RT across subjects were also removed from the behavioral analysis. This criterion resulted in the removal of 5.8% of trials.

In order to analyze the correlation of RT and accuracy with the masked priming manipulation, mixed effects models were used (Baayen et al., 2008) with RT or accuracy as the dependent variable, manipulation (prime frequency; target frequency; template match vs unmatched; root match vs unmatched) as the fixed effect, and subject and item as random intercepts. Linear mixed effects models were constructed for RT using the **lmer** function of the **lme4** package in R (Bates and Maechler, 2009), logistical mixed effects models were constructed for accuracy using the **glmer** function, and *p*-values were computed via stepwise model comparison in likelihood-ratio tests (e.g., Barr et al., 2013), such that predictors were added to the model only if they improved its overall fit. See the Supplementary Materials for additional details and full results.

##### Minimum norm estimates

MEG data were noise reduced via the Continuously Adjusted Least-Squares Method (Adachi et al., 2001), in the MEG160 software (Yokogawa Electric Corporation and Eagle Technology Corporation, Tokyo, Japan). Cortically constrained minimum-norm estimates were calculated via MNE (MGH/HMS/MIT Athinoula A. Martinos Center for Biomedical Imaging, Charleston, MA, United States) using the **mne-python** package (Gramfort et al., 2014). The cortical reconstructions were obtained using FreeSurfer (CorTechs Labs Inc., La Jolla, CA, United States and MGH/HMS/MIT Athinoula A. Martinos Center for Biomedical Imaging, Charleston, MA, United States), based on a brain template which was warped according to the head shape digitization. A source space of 5,124 points was generated for each reconstructed surface, and the boundary-element model method (BEM) was employed on activity at each source to calculate the forward solution. Using the average of all trials for a given subject, after baseline correction with the pre-target interval (-150, -50 ms)—or, equivalently, the interval (-117, -17 ms) relative to the presentation of the prime—and low pass filtering at 40 Hz, the inverse solution for this subject was computed from the forward solution in order to determine the most likely distribution of neural activity. The inverse solution was computed with a free orientation for the source estimates, meaning that the estimates were unconstrained in direction with respect to the cortical surface. MNE’s signal-to-noise ratio was set at 3.

Outlier trials including eyeblinks and excessive movements were removed based on an absolute threshold of ±2.5 pT, enforced over the time window (-100, +600 ms) for the noise reduced MEG data. In total, 33.7% of trials were discarded due to excessive noise^1^.

##### ROI analysis

FreeSurfer’s automatically parcellated anatomical regions of interest (ROIs) were used to obtain estimates of the average noise-normalized neural activity (i.e., dSPM values) within the left temporal cortical regions. Two anatomical ROIs were examined using the FreeSurfer-generated anatomical ROIs. For the M170 analysis, we investigated the effect of condition on activity in the fusiform ROI in the time window 150–250 ms post target onset. For the M350 analysis, we investigated the effect of condition on activity in the middle temporal ROI; the time window of interest was the general late interval 300–500 ms post target onset.

**FIGURE 2:**
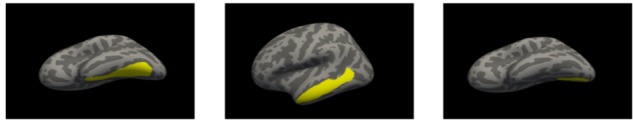
Fusiform gyrus, middle temporal ROI and posterior functional ROI.

Based on the findings of Fruchter et al. (2013), we were aware of the possibility that M170-related activity would also be found in a region posterior to the fusiform gyrus. In order to analyze this activity, a functionally defined ROI was constructed by drawing its boundaries in the common neuroanatomical space based on the grand average activation of all trials across subjects, and extracting the average dSPM values within the ROI for each subject in a “collapsed localizer” (Luck and Gaspelin, 2017). Figure 2 presents the two anatomical ROIs (fusiform gyrus and mid-temporal region) and the functional ROI (posterior fusiform gyrus).

The technique used for multiple comparisons correction was based on the methods of Maris and Oostenveld (2007), as adapted by Solomyak and Marantz (2009): we first computed Σ*t*, the sum of all *t*-values within a single temporal cluster of consecutive significant effects in the same direction (where significance is defined by |*t*| > 1.96, *p* < 0.05 uncorrected). The highest absolute value of Σ*t*, for any cluster within the whole time window, was then compared to the results of the same procedure repeated on 10,000 random permutations of the independent variable (i.e., the condition). A Monte Carlo *p*-value was thus computed, based on the percentage of times a random permutation of the independent variable led to a larger maximum absolute value of Σ*t* than the original maximum absolute value of Σ*t* (as computed on the actual data). For completeness, activation was also regressed against target frequency within the whole time windows in linear mixed effects models, with subject and item as random intercepts; see the Supplementary Materials.

### Results

#### Behavioral Results

Mean RTs and SDs for each condition are given in Table 4. The mean RTs per subject ranged from 543 to 763 ms (overall mean = 649.1 ms, median = 630.7 ms). In the Root condition, Shared Root RTs (*M* = 642 ms) were not significantly shorter than Unrelated Verb RTs (*M* = 652.9 ms), although the numerical difference (10.9 ms) is similar to that seen in previous behavioral work (Deutsch et al., 1998). RT was marginally facilitated by target frequency, χ^2^(1) = 3.5394, *p* = 0.0599. Accuracy was high, ranging from 94.7 to 99.9% (*M* = 97.8%). There were no significant differences in accuracy between any of the conditions. Full results of all statistical analyses in this paper are reproduced in the Supplementary Materials.

#### ROI Results

**FIGURE 3:**
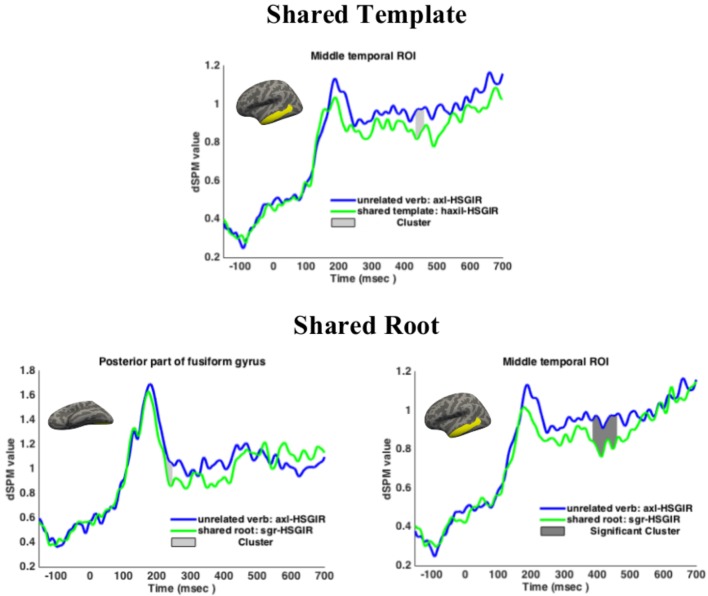
Regions of interest results for Experiment 1. Cluster-based permutation tests (Maris and Oostenveld, 2007) revealed a marginal effect of Shared Template at the M350 (top), a marginal effect of Shared Root at M170 (bottom left), and a significant effect of Shared Root at M350 (bottom right). Histograms plot mean activations per condition. Light shaded regions give the marginal clusters for the pairwise comparisons. Dark shaded regions give the significant clusters for the pairwise comparisons.

Results are presented in Figure 3. In the Shared Template condition, although the M170 showed no priming effects, the M350 did show a trend toward decreased amplitudes for template priming when compared to the unrelated control: 434–460 ms, *p* = 0.0792 (corrected over the window 300–500 ms). In the Shared Root condition, activity in the functional ROI was marginally lower than the unrelated control in the window 227–247 ms, *p* = 0.0524 (corrected over the time window 150–250 ms). Note that this cluster occurs after the M170 peak. No significant effects were found in the anatomical ROI. In the M350 a significant cluster obtained, with the Shared Root condition showing significantly lower activation than the unrelated control in the window 386–460 ms, *p* = 0.0086 (corrected over the time window 300–500 ms). This result held when adding target frequency as a predictor in a mixed effects model. Target frequency itself did not emerge as a significant predictor of activation in either of the ROIs. Priming was thus found in the M350 for the Shared Root condition and marginally so for the Shared Template condition.

### Discussion

In Experiment 1 it was expected that priming effects arise for the template *hiXYiZ* since it consists of the written prefix H- as well as an infix -I-. Following previous work on lexical access in Semitic, priming effects for the root were also expected. Given the different nature of Semitic templates to prefixes and suffixes, we did not have more specific predictions and utilized two neural correlates of lexical decomposition, the M170 and M350. Both predictions were confirmed—for the template and for the root—to different degrees.

In this experiment we aimed to replicate existing findings on root priming and template priming in an overt template using a new set of materials. The Shared Template condition showed marginal priming in the M350 component, which we take to fall in line with previous findings on affixation in English and on template priming in Hebrew (Deutsch et al., 1998). Similarly, even though the difference between Shared Template and the control failed to reach significance at the α = 0.05 level, the *p* < 0.08 trend and the waveform separation throughout the late 300–500 ms window are consistent with an affix-like role for the template, as brought out in previous work.

The lack of a finding in the M170 component was unexpected, in particular given the M350 result. As noted in our discussion of priming earlier, the M170 is sensitive to continuous factors such as transitional probability, so our binary primed/unprimed split might not have detected this effect. Another possible explanation is that the combination of a prefix and infix, taken together as one complex affix, lies beyond the low-level orthographic identification associated with the M170. However, such a view would imply that the M170 might not be sensitive to the characters comprising the discontinuous lexical root in Semitic, contrary to the trend in our current results. One additional possibility is that the combination of H- and medial -I- is ambiguous between one and two affixes: as noted in our discussion of the materials, in a word like *ha-panim* “the face” (written HPNIM), these two characters reflect two different morphemes: the definite article prefix, and a vowel which is part of the noun. In the verbs examined in Experiment 1, these characters reflect one morpheme, that of the verbal template. This ambiguity might have led to these characters not being identified exclusively as an affix, and thus not responded to by the M170. And finally, it is possible that the hypothesis regarding the M170 which stemmed from work on European languages such as English and Greek (e.g., Fruchter and Marantz, 2015; Neophytou et al., 2018) is more tightly linked to the morpho-orthographic characteristics of these languages than originally thought. The fact that the current technique did not yield unambiguous results is perhaps not surprising, given that this is the first reported experiment to use MEG for the study of morphological decomposition in non-concatenative morphology. Future work would be necessary in order to further understand the processes reflected by these neural components cross linguistically.

In the Shared Root condition, priming was found at the M350 component, in accordance with our hypothesis treating the root as a morpheme accessed during lexical retrieval. As an alternative interpretation, the M350 finding for the Shared Root condition could have presumably been due to the fact that primes in this condition had a higher degree of orthographic overlap with the target than primes in the Shared Template condition. However, it is not yet clear whether higher orthographic overlap should result in facilitation or inhibition when disentangled from an underlying morphological relation (Frost et al., 2005; Velan and Frost, 2011; Frisson et al., 2014). In order to test whether orthographic overlap had an effect on our results, two mixed effects models were constructed. One model had Overlap (in number of characters) as a by-item random effect while the other did not; no difference was found between the models, indicating that orthographic overlap did not improve the regression model’s fit to the data and hence did not influence the findings.

Behavioral priming was not found, failing to replicate previous template priming results for RTs in the template *hiXYiZ* (Deutsch et al., 1998; Frost et al., 2000a,b). One possible reason is that primes in our study appeared for 33 ms, a conservative time window, whereas those of Deutsch et al. (1998) and Frost et al. (2000a) appeared for 42 ms. Variations in SOAs lead to both qualitative (Rastle et al., 2000) and quantitative (Vorberg et al., 2004) priming effects. Additional comparison of datasets across SOAs would be needed in order to establish whether this is the reason for the failure to replicate masked priming effects behaviorally. Another possible reason is the small number of participants when compared to the behavioral literature; each of the experiments in Deutsch et al. (1998) had 96 participants and 48 target words, while ours had 21 participants and 42 target words. Bick et al. (2010) likewise found masked priming effects in fMRI for Hebrew with a relatively small number of participants but no behavioral effects in the relevant conditions. Power issues aside, we return to the issue of behavioral results in the General Discussion. Accuracy did not show sensitivity to the relevant experimental conditions in previous work.

In sum, the results for the overt template HXYIZ found priming at the M350. The root and the template were both primed, as expected yet to different extents, thereby replicating previous behavioral findings in MEG and validating our current technique. With the results for an overt template in place, we next tested a template that is not signaled overtly by the orthography.

## Experiment 2

Experiment 1 provided evidence for the claim that in an overtly affixed template, the Hebrew root and template show the same kind of priming behavior that is to be expected of morphemes in languages with concatenative morphology such as English. The results of priming an “overt” template indicate that roots and templates can both be primed, as indexed by the M350. Looking beyond Hebrew, the next experiment was designed to tease apart three possibilities regarding the mechanisms underlying masked priming: priming of overt orthographic forms, priming of abstract morphemic forms, and feature priming.

Many words in Hebrew are represented orthographically by three consonants; the examples given earlier included GDL “grew,” BSR “meat” and KTN “small.” The orthography is underspecified, in the sense that the reader must know that the string GDL is to be read *gadal* and means a verb, that the string BSR is to be read *basar* and means a noun, and that the string KTN is to be read *katan* and means an adjective. Other words are ambiguous: the string šMN could be the noun *šemen* “oil,” the adjective *šamen* “fat,” or the verb *šimen* “lubricated,” all related to the root Š-M-N. The abstract Hebrew affixes might plausibly be listed as v_CCC_ (for a verb), n_CCC_ (for a noun) and a_CCC_ (for an adjective). The consonant string CCC does not indicate on its own which of the three words is intended.

Hebrew now presents us with an added later of complexity. Previous work has indicated that the Hebrew parser treats “complex” words like *gadal* “grew” differently than “simplex” words like *basar* “meat”: in the former, the root is shared across a number of words, as explained in the Introduction. In the latter, the word does not share its root with any other nominal or verbal form. The first kind of word shows decomposition effects which the latter does not; this finding was taken to mean that readers of Hebrew utilize two parallel systems (Velan and Frost, 2011). The current study recasts this view in terms of how written words are decomposed into morphemes: what are the neural correlates of a word being decomposed into a root and a template, modeled as a root and a (possibly null) affix?

Under the model assumed thus far, decomposition occurs first, before lexical lookup. A verbal prime would be decomposed into the root and the verbal template; either of these can be used to narrow the search space for the target, leading to facilitation, i.e., priming. Experiment 2 exploited the fact that some tri-consonantal strings are nevertheless unambiguous in order to further probe lexical access. For example, the string HLX can only be read as the verb *halax* “walked.” In this case, the three consonants lead directly to the verb, decomposed into the root and the abstract form of the affix v_CCC_. If masked priming is sensitive to the visual form of overt affixes and not to covert templates, there should be no priming effect for “covert” *XaYaZ* (the root X-Y-Z plus the morpheme v_CCC_, which is null and in this case not recognized). Alternatively, if the abstract form v_CCC_ is identified despite being null, the target should then be primed. Identifying the visual form of an affix is associated with the M170 in our model; we ask whether an abstract formal representation modulates the M170 in the same way an overt formal representation does. Once the affix is identified, it is looked up, leading to M350 priming.

Finally, an even stronger prediction can be made. It has been found that in non-word strings, phonemic features show masked priming effects: the target BAF was read aloud faster when primed by *piz*, with which it shares a [labial] feature in the onset /p/, than by *suz*, whose onset /s/ is unrelated (Lukatela et al., 2001; Mousikou and Coltheart, 2014; Mousikou et al., 2015). This effect has so far not been shown for whole written forms; *typhoid* does not prime TYPHOON (Rastle et al., 2000). Certain lines of thought in the theoretical morphosyntax literature have proposed that the verb carries specific features bearing on its argument structure: a number of authors have hypothesized that a verbal construction carries one of the features [CAUSE], [DO] and [BECOME], roughly corresponding to transitive verbs, intransitive (unergative) verbs and change-of-state (unaccusative) verbs (e.g., Jackendoff, 1990; Levin and Rappaport Hovav, 1995). Folli and Harley (2006) similarly suggested that the abstract verbal morpheme itself carries one of these features, splitting our hypothetical v_CCC_ into v[+transitive]_CCC_, v[+unergative]_CCC_ and v[+unaccusative]_CCC_. If these features are part of the makeup of the verb, and if masked priming is as sensitive to features on a morpheme as it is to features on a phoneme, then there should be a priming effect for shared argument structure features in the same template. That is, transitive verbs should prime transitive verbs, activity verbs should prime activity verbs and intransitive change-of-state verbs should prime intransitive change-of-state verbs.

Three diverging predictions thus result from the intersection of the masked priming literature, the theoretical literature and the results of Experiment 1: (1) priming effects for a shared overt affix, regardless of whether the prime and target are verbs, nouns or adjectives (morphological priming is visual form priming); (2) priming effects for a shared template (morphological priming is priming of an abstract form, in this case v_CCC_); and (3) priming effects for shared argument structure (morphological priming is morphosyntactic feature priming). In fact, any priming effect for the template would constitute a novel result, as there is no orthographic difference between a shared template verb, on the one hand, and a noun with a similar syllabic shape, on the other: both are pronounced *XaYaZ* (and written XYZ).

### Methods

#### Participants

Experiment 2 was run concurrently with Experiment 1, as noted above. The same participants took part.

#### Materials

**FIGURE 4:**
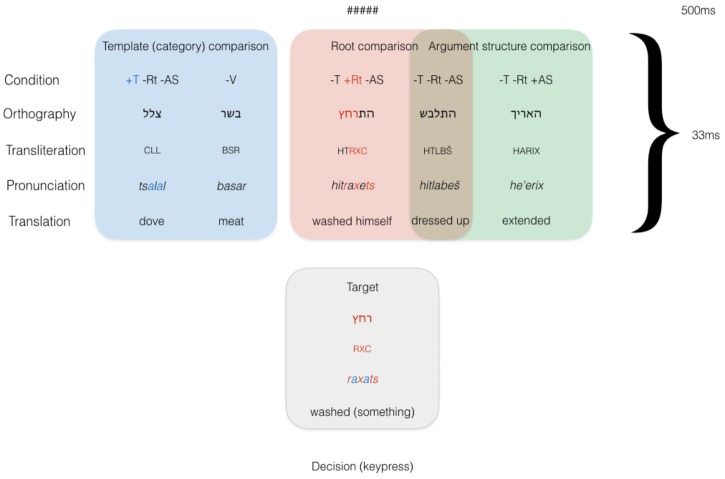
Conditions in Experiment 2.

In our priming design, the target remained constant across conditions and was preceded by five types of primes, divided into three kinds of comparisons as illustrated in Figure 4. First, a Shared Template condition was designed, similarly to Experiment 1 (+T -Rt -AS). This condition was matched with a prosodically similar, non-verbal prime (-V), in order to directly test the contrast between verbal primes on the one hand, and nominal and adjectival primes on the other. Since these nouns and adjectives are otherwise orthographically and phonologically similar, the unrelated prime controlled for potential orthographic and phonological effects. The second comparison was the Shared Root condition, again similarly to Experiment 1, in which prime and target were in different templates and either shared a root (-T +Rt -AS) or did not in an unrelated verb (-T -Rt -AS). Priming would thus only be the result of a morphological match on the root level between prime and target; semantic effects had already been ruled out for masked priming in English as well as Hebrew. Finally, the unrelated verb (-T -Rt -AS) was also employed to test the “argument structure” comparison. Hypothetically related primes were in a different template and different root, but with the same syntactic frame as the prime (-T -Rt +AS). This condition would rule in priming based on syntactic-semantic similarity: transitive verbs with transitive verbs, unergative verbs with unergative verbs, and unaccusative verbs with unaccusative verbs. Figure 4 illustrates the three pairwise comparisons. No identity condition was introduced, again in order to prevent the target item from being seen too many times throughout the experiment.

During the design, it was thought that an additional condition should be introduced, with shared argument structure in the same template (+T -Rt +AS). Were priming to obtain in this condition, however, it would be qualitatively indistinguishable from the Shared Template condition. This condition was originally included but not analyzed further as it did not contrast minimally with other conditions in an informative way.

Lexical statistics are given in Table 3. None of the differences in frequency between conditions approached significance. Any items whose frequency exceeded 500 per million were treated as outliers and excluded from the analyses. Each pair of 42 word targets and 42 non-word targets was matched with its own six possible primes, for a total of 504 items. All items were chosen such that they would be read unambiguously, as in Experiment 1. Like in Experiment 1, only word trials were included in the analysis. The order of items was pseudorandomized across participants. See the Supplementary Materials for the materials.

**Table 3:** Lexical statistics for conditions in Experiment 2.

Condition	Word length	Surface freq per million
+T -Rt -AS	3.0	11.51
-V	3.0	8.00
-T +Rt -AS	4.50	5.50
-T -Rt -AS	4.71	8.55
-T -Rt +AS	4.48	3.79
Target	3.0	10.10


#### Procedure

The procedure was identical to that of Experiment 1.

#### Analysis

Analysis methodology was identical to that in Experiment 1. The rejection rate for behavioral data was 6.4%. Argument Structure was added as a possible factor to the RT analysis. Since each target was seen six times during the experiment, there was a possible concern that participants would satiate and respond to a target much faster the more times it was presented. Therefore, Count (the number of times the specific target had been seen up to that point) was included as an additional random intercept.

The rejection rate for MEG data was 34.0%.

### Results

#### Behavioral Results

Mean RTs and SDs for each condition are given in Table 4. Mean RTs per subject ranged from 544 to 765 ms (overall mean = 648.6 ms, median = 634.1 ms). RT showed facilitation by target frequency, χ^2^(1) = 34.101, *p* < 0.0001. Accuracy was high, ranging from 87.5 to 99.6% (mean 95.1%), and was facilitated by target frequency, χ^2^(1) = 8.3959, *p* = 0.0038. There were no significant differences in accuracy between any of the conditions. There were no other behavioral results to report.

**Table 4:** Behavioral results for Experiment 2.

Condition	Words		Non-words	
		
	Mean RT	*SD*	Mean RT	*SD*
+T -Rt -AS	652.3	148.3	692.7	140.2
-T+Rt -AS	641.6	147.8	692.1	145.6
-T -Rt -AS	649.4	137.7	687.8	144.6
-T -Rt +AS	649.4	145.0	699.7	147.7
-V	654.8	145.6	691.7	154.0


#### ROI Results

**FIGURE 5:**
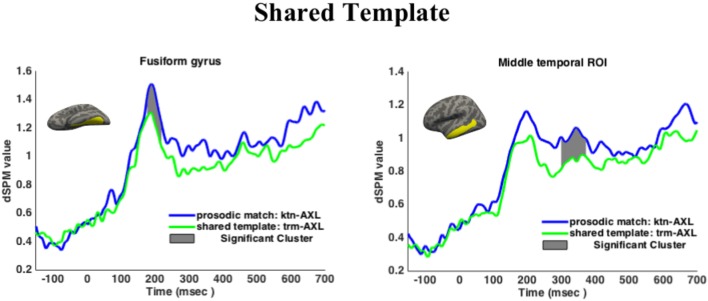
Regions of interest results for Experiment 2. Cluster-based permutation tests revealed a significant effect of Shared Template at M170 (left) and M350 (right). Histograms plot mean activations per condition. Dark shaded regions give the significant clusters for the pairwise comparisons.

Results are given in Figure 5. Shared Template showed lower activation in the anatomical ROI at M170 in the time window 177–219 ms than the prosodically matched controls, *p* = 0.0089 (corrected over the time window 150–250 ms). This result held when adding target frequency as a predictor in a mixed effects model. Shared Template also showed lower activation at M350 between 300 and 373 ms, *p* = 0.0077 (corrected over the window 300–500 ms). This result held when adding target frequency as a predictor in a mixed effects model.

Shared Root showed no difference from the control at M170 (no clusters found), at the functional ROI (no clusters found) or at M350 (no clusters found). The Argument Structure condition showed no difference from the control at M170 in either the fusiform gyrus or the functional ROI (no clusters found), nor did it differ from the control at M350 (no clusters found).

Target frequency emerged as a significant predictor of activation in both ROIs for Experiment 2 in mixed effect models, for the fusiform gyrus in the time window 150–250 ms (χ^2^(1) = 11.527, *p* = 0.0068) and for the middle temporal region in the time window 300–500 ms (χ^2^(1) = 6.9125, *p* = 0.00856). When target frequency was added as a predictor in the pairwise comparisons as reported above, to mixed effects models fitted over the time windows, no differences were found in the overall result. That is to say, none of the results reported above were driven by frequency. Priming was thus observed for the Shared Template condition but not for the Shared Root or Shared Argument Structure conditions.

### Discussion

Neural activation was lower at both the M170 and M350 components when the prime and target shared the “covert” XYZ template, in effect revealing a situation of category priming (verbs primed verbs, but non-verbs did not prime verbs). This result follows if the abstract visual form of an affix is what was primed, the affix itself also containing categorial information. It is striking that this priming effect obtains, given that the Shared Template condition and the control were not immediately different in any other way: their orthographic, phonological and prosodic shapes are similar (XYZ in the orthography, *XaYaZ* in pronunciation). The only distinction is between verbs in a given template on the one hand (Shared Template) and non-verbs on the other (control condition). The fact that masked priming has obtained in general between verbal templates but not nominal patterns (with the exception of recent studies such as Deutsch et al., 2016) is likely related to there existing seven verbal templates but numerous nominal patterns. This finding supports a role for abstract verbalizing morphemes in the internal structure of words, even in languages with non-concatenative morphology.

Root priming did not obtain, perhaps surprisingly given the previous work surveyed earlier. This null result is not consistent with our overall predictions. Recall, however, that were this result to obtain it would have been novel: root priming in Hebrew verbs has only been shown to arise in templates marked by overt affixes (like that in Experiment 1), in pseudo-words and in some “weak” roots such as those containing glides (Deutsch et al., 1998; Frost et al., 2000a; Velan et al., 2005). Farhy et al. (2017) similarly failed to find a priming effect in XYZ, attributing this null result to the lower productivity of the template, but that explanation does not accord with the template priming results reported here. This pattern remains a puzzling one for studies of lexical access in Hebrew as well as cross linguistically.

Returning to the model proposed by Velan and Frost (2011), it has been claimed that some nouns are not decomposed into a root and a pattern, since the root cannot be seen in any other word of the language. In our materials, all verbs were “decomposable” under this definition, although the verbal templates might enjoy privileged structure (either because there are only a few of them, or because verbal templates are associated with different syntactic frames). Nevertheless, the possibility remains that our findings reflect a fundamental difference between words whose root is instantiated in other forms, and those that do not; our own materials were divided roughly equally between these two classes. If this alternative hypothesis were correct, the prediction would be that words sharing a root would show priming effects since they are decomposed, whereas words not sharing a root with other words would not be decomposed and hence not primed. In order to rule out this possibility, two *post hoc* analyses were conducted.

First, a pairwise comparison was performed between the –T–Rt–AS condition and the -V condition. Items in the former condition were verbal forms in one of the other overt templates, all “decomposable” in the current sense. On our hypothesis they are not predicted to show a priming effect (no relationship between prime and target), while on the alternative hypothesis being currently discussed they should (the “decomposable” target items lead to priming, as in the +T condition). The latter prediction was not borne out: In the M350, where a robust pattern was found for +T, no cluster was found for –T–Rt–AS. In the M170, a marginal window was found which was both much shorter than the +T comparison (178–195 ms, as opposed to 177–219 ms for +T) and much less robust (*p* = 0.08, as opposed to *p* < 0.01). This overall null result does not rule out an explanation in terms of “decomposability” completely but it does call it into question, whereas this non-finding is not problematic for our own model.

Therefore, in our second follow-up the MEG analysis was run again, this time binning the items in the -V condition in two bins: those that are “decomposable” in the current sense and those that are not. The results showed no difference that is attributable to this distinction. Accordingly, we find no evidence for the alternative hypothesis, at least until it can be tested in a more targeted manner in future work. For example, as larger corpora become available for languages like Hebrew, it would be important to test whether these “non-decomposable” nouns show distinct form typicality effects than verbs, as recent work has suggested for the formal properties of nouns and verbs in English (Sharpe and Marantz, 2017). That is to say, perhaps the phonological properties of these two word classes can be distinguished, in which case an alternative source of information would be available to the reader. In the meantime—and in line with these recent findings—the distinction between the two kinds of words has been translated into a model in which all words are decomposed. What the current technique would enable us to do in the future is track this decomposition neurophysiologically: a higher degree of decomposition would be reflected by a stronger priming effect (Fruchter et al., 2013).

## General Discussion

Research on morphological decomposition often capitalizes on the distinction between stems and affixes. The former are taken to contain lexical information while the latter are considered to be more functional. The behavior of each can be evaluated separately (Rastle et al., 2000) or combined in order to probe their interaction, especially in irregular verbs (Crepaldi et al., 2010; Fruchter et al., 2013). Yet work on decomposition has often limited itself to European languages, save for the notable exceptions mentioned earlier. These studies indicated that in Semitic languages it is possible to equate the root with a stem and the template with an affix, at least as a first approximation.

Two verbal templates were at the focus of the current study. For the “overtly” marked verbal template of Experiment 1, it was found that primes which share the template and primes which share the root lead to lower M350 activation on the target, consistent with previous behavioral findings. For the “covertly” marked verbal template of Experiment 2, it was found that a verb also primes a target in the same template, as indexed by the M170 and M350. Potential confounds such as frequency and orthographic overlap did not have an effect.

Even though our study led to a number of neurophysiological findings, no behavioral results were found between conditions. We have discussed a number of reasons for why existing behavioral results were not replicated in Experiment 1 and for why MEG but not behavioral results obtained in Experiment 2, but it is important to consider possible differences between MEG results and behavioral ones in general. As noted in the Introduction, it has been argued that a lexical decision task taps into more than just comprehension, introducing another cognitive step influencing RT (Marelli et al., 2013). The advantage of techniques like MEG is that they allow for fine-grained temporal resolution, enabling researchers to study “early” effects of lexical decomposition (Zweig and Pylkkänen, 2009). A situation in which MEG findings coexist with a null behavioral finding is therefore not unlikely, if further downstream effects influence the behavioral results (Stockall and Marantz, 2006). This point emphasizes the need for explicit models of lexical access which identify different stages of processing (Marelli et al., 2013; Fruchter and Marantz, 2015). We have assumed that reduced activation as a result of priming in lexical decision is a qualitatively similar phenomenon in both MEG and RT. Elucidating a model which correctly predicts results across different investigative techniques remains an important goal for work in the field.

The current study asked what roots and templates can be primed, and by implication what a morpheme is: is it the form plus a meaning? Just the form? Or is it something more abstract, such as an abstract form or a bundle of grammatical features? The hypothesis consistent with the findings is that the abstract visual form of an affix can be recognized at the stage in which visual morphological forms are processed, as indexed by the M170. However, the null affix was only recognized if its existence as part of the stimulus was unambiguous. In Experiment 2 it was found that an abstract representation of verbal morphology can be covert: on seeing the Hebrew string ZKR (for *zaxar* “remembered”), the reader recovers the lexical category. Here there are only a few options, namely noun, verb or adjective (perhaps preposition as well). Sensitivity to verbal primes but not to comparable nominal or adjectival primes challenges the purely overt formal hypothesis and supports a more abstract representation of the morpheme. In contrast, on seeing the string HZKIR (*hizkir* “reminded”) as in Experiment 1, the reader might immediately recognize the prefix H- and infix -I-, identifying the string as a verbal form and engaging in root lookup. A more conventional hypothesis, according to which only overt visual forms are primed, is incompatible with the findings of Experiment 2, as is a hypothesis relating priming to the underlying syntactic features of a morpheme. The feature hypothesis is also the closest that one can come to meaning-based priming within this experimental paradigm, since masked priming in a 33 ms SOA does not trigger meaning lookup. Semitic languages are a good testing ground for studies of lexical access for two reasons: the decomposition is often non-linear, and the affix, so to speak, might not be visible in the orthography at all (as in Experiment 2). The fact that root and template priming can be shown to obtain indicates that a nuanced view of the morpheme as the basic compositional unit needs to be adopted, including abstract, “covert” affixes.

In conclusion a specific view of morphological priming was tested. When priming the English prefix *un-* as in *unfair* (Chateau et al., 2002), we might ask whether what is primed is orthographic “un-” or abstract [NEGATIVE ADJECTIVE]. In Hebrew there are many such affixes, taking the form of morphophonological patterns: HXYIZ is both a single form and a collection of morphemes. XYZ is both a single form and potentially a verb in the template *XaYaZ*, a noun or an adjective. Future studies will need to test whether these results generalize across additional materials, in Hebrew as well as other Semitic languages. In this context, it can be noted that recent work has begun to identify how readers identify whether ambiguous strings like *hammer* correspond to a verb or a noun (King et al., 2015; Sharpe and Marantz, 2017). It remains to be seen whether the results of the current study can be explained by appealing to different form typicality effects for nouns and verbs, and whether languages like English show the same priming patterns as Hebrew when a null morpheme can be recognized unambiguously.

## Ethics Statement

This study was carried out in accordance with the recommendations of the Institutional Review Board of New York University. The protocol was approved by the Institutional Review Board of New York University. All subjects gave written informed consent in accordance with the Declaration of Helsinki.

## Author Contributions

IK, LP, and AM designed the study. IK ran the study and performed the analysis. IK drafted the work. All authors critically revised the drafts.

## Conflict of Interest Statement

The authors declare that the research was conducted in the absence of any commercial or financial relationships that could be construed as a potential conflict of interest.

## Footnotes

1 This relatively high rejection rate was due to noisy recording conditions in our New York facility. The Supplementary Materials list rejection rates by condition.
