# Supplementary material for: The Form of Morphemes: MEG Evidence From Masked Priming of Two Hebrew Templates
Source: Front Psychol. 2018 Nov 12;9:2163. doi: 10.3389/fpsyg.2018.02163 (PMC6240614; doi:10.3389/fpsyg.2018.02163)
Supplement: Supplementary file 1 [file Data_Sheet_1.DOCX]

Supplementary Material

The form of morphemes: MEG evidence from masked priming of two Hebrew templates

Itamar Kastner*, Liina Pylkkänen, Alec Marantz

*** Correspondence:** Itamar Kastner: itamar@itamarkast.net

# Materials: Experiment 1

| **Primes** | | | | | |
| --- | --- | --- | --- | --- | --- |
| **+T -Rt** | | | **-T +Rt** | | |
| **phonology** | **translation** | **orthography** | **phonology** | **translation** | **orthography** |
| higdil | enlarged | hgdil | da'ag | worried | dag |
| hirgi'a | calmed | hrgi' | hit'arex | grew long | htark |
| histir | hid | hsTir | nizhar | was careful | nzhr |
| hiSbiax | improved | hSbix | nixnas | entered | nkns |
| hiStik | silenced | hStiq | amad | stood | 'md |
| higdir | defined | hgdir | hitxabe | hid | htxba |
| hixSiv | considered | hxSiv | nizkar | remembered | nzkr |
| he'enik | granted | h'niq | nitma | was assimilated | nTm' |
| hitrid | bothered | hTrid | histaber | turned out | hstbr |
| he'evir | passed along | h'bir | lavaS | wore | lbS |
| hicxik | made laugh | hcxik | paka | burst | pq' |
| hiSlix | tossed away | hSlix | hitkarev | came close | htqrb |
| hidlik | lit up | hdliq | hitbalet | stood out | htblT |
| hiS'il | lent | hSail | nizrak | was thrown | nzrq |
| hicliax | succeeded | hclix | para | paid off | pr' |
| hitbia | sunk | hTbi' | hitbareg | found his place | htbrg |
| hiSmi'a | played a sound | hSmi' | barax | flee | brx |
| he'exil | feed | haxil | nig'al | was repulsed | ng'l |
| hit'in | charge | hT'in | hitgaSem | came true | htgSm |
| hiv'ir | set alight | hb'ir | nidham | was amazed | ndhm |
| hiStin | urinated | hStin | nexlat | was strained (leaf) | nxlT |
| he'esik | employed | h'siq | huxram | was boycotted | huxrm |
| hirSim | impressed | hrSim | nilxac | be pressured, pressed | nlxc |
| hixkir | leased | hxkir | hitmace | was oriented | htmca |
| hiflil | incriminate | hplil | hitnaceax | argued | htncx |
| hicliv | crossed | hclib | nisgar | closed | nsgr |
| hix'is | angered | hk'is | nispag | was soaked up | nspg |
| hiznik | started off | hzniq | ne'elav | be insulted | n'lb |
| hifxit | lessened | hpxit | hitpaSet | undressed | htpST |
| hixpil | multiplied | hkpil | hucnax | was parachuted | hucnx |
| hinxit | landed | hnxit | ratax | boil | rtx |
| hicmiax | grew | hcmix | niS'ar | remain | nSar |
| hifxid | frightened | hpxid | tasas | fermented, bubbled | tss |
| hir'iS | made noise | hr'iS | taram | donate | trm |
| hicbi'a | voted, pointed | hcbi' | hitmaten | mellowed | htmtn |
| hismik | blushed | hsmiq | hit'aper | applied make up | htapr |
| hivdil | differentiated | hbdil | hiStake'a | settled down | hStq' |
| hidbir | exterminated | hdbir | huksam | was charmed | huqsm |
| hivhil | scare | hbhil | kafa | froze | kpa |
| higbir | amplified | hgbir | hitraxev | expanded | htrxb |
| hisni | cause to hate | hsnia | ho'aSam | was blamed | hoaSm |
| hiSpil | humiliated | hSpil | nidrax | became alert | ndrx |

| **Primes** | | | **Target** | | | **Nonword** |
| --- | --- | --- | --- | --- | --- | --- |
| **-T -Rt** | | |  |  |  |  |
| **phonology** | **translation** | **orthography** | **phonology** | **translation** | **orthography** | **orthography** |
| baxa | cried | bkh | hid'ig | caused to worry | hdaig | hrkis |
| gasas | died out | gss | he'erix | lengthened | harix | hSliq |
| nataf | dripped | nTp | hizhir | warned | hzhir | hgdiq |
| kara | happened | qrh | hexnis | put in | hxnis | hrhiz |
| tafax | swelled up | tpx | he'emid | stood up | h'mid | hsnim |
| nafal | fell | npl | hixbi | hide | hxbia | hrcix |
| xalaf | passed | xlp | hizkir | reminded | hzkir | hdmir |
| da'ax | petered out | d'k | hitmi'a | immersed | hTmi' | hnbix |
| hiStabeS | went wrong | hStbS | hisbir | explained | hsbir | hq'ip |
| haya | was | hih | hilbiS | dressed up | hlbiS | hmTid |
| hitgared | scratched | htgrd | hifki'a | confiscated | hpki' | hlSib |
| hit'alef | fainted | ht'lp | hikriv | sacrificed | hqriv | hlgiz |
| nextaf | was snatched | nktp | hivlit | made stand out | hbliT | hbrik |
| nilxac | was pressured | nlxc | hizrik | injected | hzrik | hTlib |
| nisgar | was closed | nsgr | hifri'a | interrupted | hpri' | hzqil |
| ta'a | be wrong | T'h | hivrig | screwed | hbrig | hr'iq |
| yarad | descend | ird | hivriax | chased away/smuggled | hbrix | hgmiz |
| nadad | wandered | ndd | hig'il | grossed out | hg'il | hrbig |
| calal | dove | cll | higSim | made come true | hgSim | hlaiS |
| navax | barked | nbx | hidhim | amazed | hdhim | hSmig |
| natan | give | ntn | hixlit | decided | hxliT | hmaiS |
| saxa | swam | sxh | hixrim | confiscated, boycotted | hxrim | hlTib |
| canax | parachute | cnx | hilxic | pressured | hlxic | hrSix |
| nasa | traveled | ns' | himci | invented | hmcia | hcxil |
| hit'amen | train | htamn | hinciax | commemorated | hncix | hsmiT |
| hitbadeax | joked around | htbdx | hisgir | turned in | hsgir | hxnic |
| hitgaleax | shaved | htglx | hispig | made soak up | hspig | hrsig |
| hitxaret | regret | htxrT | he'eliv | insulted | h'lib | hsmig |
| rakad | dance | rqd | hifSit | undressed | hpSiT | hb'id |
| hityabeS | dry up, dehydrate | htibS | hicniax | parachuted | hcnix | hSmip |
| hitmaxa | specialize | htmxh | hirtiax | boiled | hrtix | hgdim |
| hitkavec | shrink | htqvc | hiS'ir | left sth | hSair | hxTiT |
| nivzaz | be pillaged | nbzz | hitsis | instigated | htsis | hdmiS |
| pizer | scattered | pzr | hitrim | fundraised | htrim | hstit |
| ti'ev | despise | t'b | himtin | wait | hmtin | hrTim |
| citet | quote | cTT | he'efir | greyed | hapir | hnmiT |
| za'ak | shout | z'q | hiSkia | invest | hSqi' | hb'ig |
| histaper | get a haircut | hstpr | hiksim | charmed | hqsim | hS'iq |
| hises | hesitate | hss | hikpi | froze | hkpia | hkmis |
| hiSta'el | cough | hSt'l | hirxiv | expanded | hrxib | hp'iT |
| hitxamek | evade | htxmk | he'eSim | blamed | haSim | hbxiS |
| hitpale | be puzzled | htpla | hidrix | guided | hdrix | htlis |

# Materials: Experiment 2

|  | **Primes** | | | | | | | |
| --- | --- | --- | --- | --- | --- | --- | --- | --- |
|  | **+T -Rt -AS** | | | | **-V** | | | |
|  | **phonology** | **translation** | **features** | **orthography** | **phonology** | **translation** | **category** | **orthography** |
|  | calal | dove | unergative | cll | basar | meat | noun | bsr |
|  | galaS | surfed, spilled | intransitive | glS | maxar | tomorrow | adverb | mxr |
|  | Saga | was mistaken | unergative | Sgh | kahal | audience | noun | qhl |
|  | xasaf | exposed | transitive | xsp | ganan | gardener | noun | gnn |
|  | zarak | threw | transitive | zrq | matan | giving | noun | mtn |
|  | ta'an | load, argue | transitive | T'n | hadar | glory | noun | hdr |
|  | xaca | cross, cut | transitive | xch | zahav | gold | noun | zhb |
|  | navax | barked | unergative | nbx | Sa'an | watchmaker | noun | S'n |
|  | asa | did | transitive | sh | kanaf | wing | noun | knp |
|  | ga'al | redeemed | transitive | gal | xaxam | smart | adjective | xkm |
|  | saxav | carried | transitive | sxb | maca | matzoh | noun | mch |
|  | bala | swallowed | transitive | bl' | Safan | hare | noun | Spn |
|  | na'am | gave a speech | unergative | nam | nahar | river | noun | nhr |
|  | gasas | was dying | unaccusative | gss | adam | man | noun | adm |
|  | sarar | reign, be | unaccusative | srr | barad | hail | noun | brd |
|  | dahar | galloped | unergative | dhr | panas | flashlight | noun | pns |
|  | za'ak | shouted | unergative | z'q | barad | hail | noun | brd |
|  | gava | died out | unaccusative | gb' | magaf | boot | noun | mgf |
|  | carax | screamed | unergative | crx | sapa | sofa | noun | sph |
|  | acar | curated | transitive | 'cr | pagaz | mortar shell | noun | pgz |
|  | ta'a | deviated | unergative | t'h | dagan | cereal | noun | dgn |
|  | zarax | rose | unergative | zrx | masa | journey | noun | ms' |
|  | ratan | grunted | unergative | rTn | balaS | detective | noun | blS |
|  | da'ax | petered out | unaccusative | d'k | makak | cockroach | noun | mqq |
|  | bara | created | transitive | bra | tamar | date (fruit) | noun | tmr |
|  | nagax | headbutted | transitive | ngx | safam | mustache | noun | spm |
|  | haya | was | copula | hih | nadal | centipede | noun | ndl |
|  | alaz | rejoiced | unergative | 'lz | afar | dirt | noun | 'pr |
|  | halax | went | intransitive | hlx | calaf | sniper | noun | clp |
|  | rakax | concocted | transitive | rqx | magaS | tray | noun | mgS |
|  | para | paid off | transitive | pr' | xagav | grasshopper | noun | xgb |
|  | daraS | demanded | transitive | drS | nagar | carpenter | noun | ngr |
|  | arav | ambushed | unergative | arb | raban | rabbi | noun | rbn |
|  | naxar | snored | unergative | nxr | aSan | smoke | noun | 'Sn |
|  | Sa'at | stampeded | unergative | S'T | agam | lake | noun | agm |
|  | nataf | dripped | unaccusative | nTp | tanax | bible | noun | tnx |
|  | kara | happened | unaccusative | qrh | anan | cloud | noun | 'nn |
|  | amad | stood up | intransitive | 'md | raxav | wide | adjective | rkb |
|  | caram | grated | intransitive | crm | magal | sickle | noun | mgl |
|  | alac | rejoiced | unergative | 'lc | anak | giant/necklace | noun | 'nq |
|  | Sa'ag | roared | unergative | Sag | masa | load | noun | msa |
|  | mahal | diluted | ditransitive | mhl | para | cow | noun | prh |

# Statistical tests

## ROI results: Cluster permutation tests

### Experiment 1

#### Shared Template, M170

(No clusters found)

#### Shared Template, M350

| p-value | Cluster | +T Mean | +T SE | +V Mean | +V SE |
| --- | --- | --- | --- | --- | --- |
| 0.0792 | 434-460ms | 1.001 | 0.074 | 0.871 | 0.056 |

#### Shared Root, M170 functional ROI

| p-value | Cluster | +Rt Mean | +Rt SE | +V Mean | +V SE |
| --- | --- | --- | --- | --- | --- |
| 0.0524 | 227-274ms | 1.087 | 0.053 | 0.918 | 0.063 |

#### Shared Root, M350

| p-value | Cluster | +Rt Mean | +Rt SE | +V Mean | +V SE |
| --- | --- | --- | --- | --- | --- |
| 0.0086 | 386-460ms | 0.978 | 0.07 | 0.828 | 0.053 |

### Experiment 2

#### Shared Template, M170

| p-value | Cluster | +T Mean | +T SE | –V Mean | –V SE |
| --- | --- | --- | --- | --- | --- |
| 0.0089 | 177-219ms | 1.417 | 0.128 | 1.235 | 0.11 |

#### Shared Template, M350

| p-value | Cluster | +T Mean | +T SE | –V Mean | –V SE |
| --- | --- | --- | --- | --- | --- |
| 0.0077 | 300-373ms | 1.039 | 0.086 | 0.885 | 0.063 |

#### Shared Root, M170

(No clusters found)

#### Shared Root, M350

(No clusters found)

#### Shared Argument Structure, M170

(No clusters found)

#### Shared Argument Structure, M350

(No clusters found)

## Behavioral results: Mixed effects models

Frequency was first tested as a main effect in each experiment. If it was significant (or even marginally significant at *p* < 0.1), it was included as a predictor in the planned pairwise comparisons between conditions.

For example, in order to assess the effect of Shared Root (+Rt) on RT in 3.2.1.3.1:

model.baseline <- lmer(RT ~ (1 | item) + (1 | subject), data = Exp1)
model.frequency <- lmer(RT ~ target_frequency + (1 | item) + (1 | subject), data = Exp1)
anova(model.baseline, model.frequency)
# chisq(1) = 3.5046, p = 0.0612
model.full <- lmer(RT ~ target_frequency + condition + (1 | item) + (1 | subject), data = Exp1)
anova(model.frequency, model.full)
# chisq(1) = 1.5934, p = 0.2068

Similarly, the basic formula for Experiment 2 included Count, as explained in the Analysis section of Experiment 2 in the main text:

model <- lmer(RT ~ (1 | item) + (1 | subject) + (1 | count), data = Exp2)

Note further that mixed effects models are computed over individual trials, unlike the MEG cluster permutation tests. The latter are calculated using averages over trials in a region, as mentioned in the main text.

### Experiment 1

#### RT ~ Target Frequency

| Predictor | Estimate | SE | t-value | p-value (χ^2^) |
| --- | --- | --- | --- | --- |
| (Intercept) | 657.0501 | 18.8572 | 34.84 |  |
| Target Frequency | -0.5037 | 0.2670 | 1.89 | 0.05993 |

Observations: 2,445. Var(Item): 681.1. Var(Subject): 7189.0.

#### Accuracy ~ Target Frequency

| Predictor | Estimate | SE | z-value | p-value (χ^2^) |
| --- | --- | --- | --- | --- |
| (Intercept) | 4.1225 | 0.273 | 15.1 |  |
| Target Frequency | 0.1176 | 0.157 | 0.75 | 0.418 |

Observations: 2,445. Var(Item): 0.562. Var(Subject): 0.202.

#### RT ~ Condition

##### RT ~ Shared Root

| Predictor | Estimate | SE | t-value | p-value (χ^2^) |
| --- | --- | --- | --- | --- |
| (Intercept) | 680.8618 | 27.9534 | 24.357 |  |
| Target Frequency | -0.5995 | 0.3165 | 1.894 | 0.0612 |
| Condition | -10.1437 | 8.0995 | 1.252 | 0.2068 |

Observations: 1,635. Var(Item): 591.6. Var(Subject): 7,408.3.

##### RT ~ Shared Template

| Predictor | Estimate | SE | t-value | p-value (χ^2^) |
| --- | --- | --- | --- | --- |
| (Intercept) | 662.5264 | 22.8376 | 29.010 |  |
| Target Frequency | -0.6361 | 0.3467 | 1.835 | 0.0659 |
| Condition | -0.9897 | 8.9370 | 0.111 | 0.9113 |

Observations: 1,629. Var(Item): 897.2. Var(Subject): 6,696.5.

#### Accuracy ~ Condition

##### Accuracy ~ Shared Root

| Predictor | Estimate | SE | z-value | p-value (χ^2^) |
| --- | --- | --- | --- | --- |
| (Intercept) | 4.062 | 0.395 | 10.3 |  |
| Condition | 0.5588 | 0.4298 | 1.3 | 0.194 |

Observations: 1,635. Var(Item): 0.638. Var(Subject): 0.080.

##### Accuracy ~ Shared Template

| Predictor | Estimate | SE | z-value | p-value (χ^2^) |
| --- | --- | --- | --- | --- |
| (Intercept) | 4.0883 | 0.3552 | 11.509 |  |
| Condition | -0.212 | 0.3512 | 0.604 | 0.546 |

Observations: 1,629. Var(Item): 0.417. Var(Subject): 0.316.

### Experiment 2

#### RT ~ Target Frequency

| Predictor | Estimate | SE | t-value | p-value (χ^2^) |
| --- | --- | --- | --- | --- |
| (Intercept) | 659.0175 | 19.5683 | 33.68 |  |
| Target Frequency | -0.6652 | 0.1103 | 6.03 | < 0.0001 |

Observations: 4,920. Var(Item): 1,041.5. Var(Subject): 6,729.1. Var(Count): 327.9.

#### Accuracy ~ Target Frequency

| Predictor | Estimate | SE | z-value | p-value (χ^2^) |
| --- | --- | --- | --- | --- |
| (Intercept) | 3.609 | 0.244 | 14.798 |  |
| Target Frequency | 0.0212 | 0.0066 | 3.211 | 0.00031 |

Observations: 4,920. Var(Item): 1.236. Var(Subject): 0.546. Var(Count): 0.060.

#### RT ~ Condition

##### RT ~ Shared Root

| Predictor | Estimate | SE | t-value | p-value (χ^2^) |
| --- | --- | --- | --- | --- |
| (Intercept) | 650.2741 | 21.1146 | 30.797 |  |
| Condition | 2.2559 | 2.1784 | 1.036 | 0.2959 |
| Target Frequency | -0.6532 | 0.1836 | 3.558 | 0.00056 |

Observations: 1,630. Var(Item): 936.3. Var(Subject): 6,850.0. Var(Count): 326.4.

##### RT ~ Shared Template

| Predictor | Estimate | SE | t-value | p-value (χ^2^) |
| --- | --- | --- | --- | --- |
| (Intercept) | 660.9248 | 24.2475 | 27.257 |  |
| Target Frequency | -0.310 | 0.1841 | 3.873 | 0.000173 |
| Condition | 0.9033 | 2.9144 | 0.310 | 0.7534 |

Observations: 1,629. Var(Item): 910.9. Var(Subject): 7,264.4. Var(Count): 285.9.

##### RT ~ Shared Argument Structure

| Predictor | Estimate | SE | t-value | p-value (χ^2^) |
| --- | --- | --- | --- | --- |
| (Intercept) | 653.4343 | 46.4195 | 14.077 |  |
| Target Frequency | -0.7901 | 0.1985 | 3.980 | 0.0001182 |
| Condition | 1.8455 | 9.4183 | 0.196 | 0.843 |

Observations: 1,645. Var(Item): 1,216.7. Var(Subject): 6,091.9. Var(Count): 260.4.

#### Accuracy ~ Condition

##### Accuracy ~ Shared Root

| Predictor | Estimate | SE | z-value | p-value (χ^2^) |
| --- | --- | --- | --- | --- |
| (Intercept) | 3.8058 | 0.4525 | 8.41 |  |
| Target Frequency | 0.0283 | 0.0146 | 1.944 | 0.01648 |
| Condition | -0.0632 | 0.1013 | 0.624 | 0.533 |

Observations: 1,630. Var(Item): 1.512. Var(Subject): 0.31. Var(Count): <0.0001.

##### Accuracy ~ Shared Template

| Predictor | Estimate | SE | z-value | p-value (χ^2^) |
| --- | --- | --- | --- | --- |
| (Intercept) | 3.4648 | 0.603 | 5.747 |  |
| Target Frequency | 0.0177 | 0.0099 | 1.782 | 0.04907 |
| Condition | 0.02776 | 0.1137 | 0.244 | 0.9651 |

Observations: 1,629. Var(Item): 0.928. Var(Subject): 0.748. Var(Count): 0.072.

##### Accuracy ~ Shared Argument Structure

| Predictor | Estimate | SE | z-value | p-value (χ^2^) |
| --- | --- | --- | --- | --- |
| (Intercept) | 3.1931 | 1.662 | 1.921 |  |
| Target Frequency | 0.1008 | 0.0436 | 2.31 | <0.0001 |
| Condition | 0.0269 | 0.3627 | 0.074 | 0.941 |

Observations: 1,645. Var(Item): 1.084. Var(Subject): 0.638. Var(Count): <0.0001.

## ROI results: Mixed effects models

Frequency was first tested as a main effect in each experiment. If it was significant (or marginally significant), it was included as a predictor in the planned pairwise comparisons between conditions.

These models are included for completeness; M170 results are for the entire window 150-250ms, and M350 results for the entire window 300-500ms, unlike the more fine-grained cluster permutation tests reported in the main text.

### Experiment 1

#### Activation ~ Target Frequency

##### M170 ~ Target Frequency

| Predictor | Estimate | SE | t-value | p-value (χ^2^) |
| --- | --- | --- | --- | --- |
| (Intercept) | 1.696203 | 0.036013 | 47.10 |  |
| Target Frequency | -0.001155 | 0.001327 | 0.87 | 0.3834 |

Observations: 1,754. Var(Item): 0.0009. Var(Subject): 0.0202.

##### M350 ~ Target Frequency

| Predictor | Estimate | SE | t-value | p-value (χ^2^) |
| --- | --- | --- | --- | --- |
| (Intercept) | 1.6921688 | 0.0508596 | 33.27 |  |
| Target Frequency | -0.0009727 | 0.0013301 | 0.73 | 0.4641 |

Observations: 1,754. Var(Item): 0.0014. Var(Subject): 0.0472.

#### Activation ~ Condition

##### M170 ~ Shared Template

| Predictor | Estimate | SE | t-value | p-value (χ^2^) |
| --- | --- | --- | --- | --- |
| (Intercept) | 1.65657 | 0.06895 | 24.027 |  |
| Condition | -0.02946 | 0.03966 | 0.743 | 0.4578 |

Observations: 1,169. Var(Item): <0.0001. Var(Subject): 0.0172.

##### M350 ~ Shared Template (0.0792)

| Predictor | Estimate | SE | t-value | p-value (χ^2^) |
| --- | --- | --- | --- | --- |
| (Intercept) | 1.61567 | 0.08169 | 19.778 |  |
| Condition | -0.05912 | 0.04282 | 1.381 | 0.1675 |

Observations: 1,169. Var(Item): 0.0023. Var(Subject): 0.0437.

##### M350 ~ Shared Template + (1 + Overlap | Item)

| Predictor | Estimate | SE | t-value | p-value (χ^2^) |
| --- | --- | --- | --- | --- |
| (Intercept) | 1.6042 | 0.0864 | 18.574 |  |
| Condition | 0.0633 | 0.0451 | 1.403 | (0.1675) |

Observations: 1,169. Var(Item): 0.0044. Var(Overlap | Item): 0.0045. Var(Subject): 0.0442.

##### M170 ~ Shared Root

| Predictor | Estimate | SE | t-value | p-value (χ^2^) |
| --- | --- | --- | --- | --- |
| (Intercept) | 1.80994 | 0.11285 | 16.039 |  |
| Condition | -0.04741 | 0.04301 | 1.103 | 0.2679 |

Observations: 1,166. Var(Item): 0.0006. Var(Subject): 0.0147.

##### M350 ~ Shared Root + Target Frequency

χ^2^(1) = 0.0165, *p* = 0.8979 for the model with Target Frequency added.

| Predictor | Estimate | SE | t-value | p-value (χ^2^) |
| --- | --- | --- | --- | --- |
| (Intercept) | 1.8914401 | 0.1151891 | 16.420 |  |
| Condition | -0.0791051 | 0.0408679 | 1.936 | 0.05277 |
| Target Frequency | -0.0002033 | 0.0015410 | 0.132 | 0.4641 |

Observations: 1,166. Var(Item): <0.0001. Var(Subject): 0.0049.

##### M350 ~ Shared Root + (1 + Overlap | Item)

| Predictor | Estimate | SE | t-value | p-value (χ^2^) |
| --- | --- | --- | --- | --- |
| (Intercept) | 1.8904 | 0.1149 | 16.455 |  |
| Condition | -0.0791 | 0.0409 | 1.936 | (0.05277) |

Observations: 1,166. Var(Item): <0.0001. Var(Overlap | Item): <0.0001. Var(Subject): 0.0049.

### Experiment 2

#### Activation ~ Target Frequency

##### M170 ~ Target Frequency

| Predictor | Estimate | SE | t-value | p-value (χ^2^) |
| --- | --- | --- | --- | --- |
| (Intercept) | 1.6997295 | 0.0376744 | 45.12 |  |
| Target Frequency | 0.0018531 | 0.0005418 | 3.42 | 0.0068 |

Observations: 3,494. Var(Item): 0.0017. Var(Subject): 0.0256.

##### M350 ~ Target Frequency

| Predictor | Estimate | SE | t-value | p-value (χ^2^) |
| --- | --- | --- | --- | --- |
| (Intercept) | 1.7149417 | 0.0491395 | 34.90 |  |
| Target Frequency | 0.0016183 | 0.0006153 | 2.63 | 0.00856 |

Observations: 3,494. Var(Item): <0.0001. Var(Subject): 0.0453.

#### Activation ~ Condition

##### M170 ~ Shared Template + Target Frequency

χ^2^(1) = 10.866, *p* = 0.0009795 when adding Condition to Frequency.

The interaction does not speak to any specific hypothesis and was not interpreted. Informally, it would indicate that low-frequency non-verb targets showed greater priming effects.

| Predictor | Estimate | SE | t-value | p-value (χ^2^) |
| --- | --- | --- | --- | --- |
| (Intercept) | 1.530049 | 0.081317 | 18.816 |  |
| Target Frequency | -0.002675 | 0.002795 | 0.957 | < 0.0001 |
| Condition | 0.030600 | 0.015251 | 2.006 | 0.0009795 |
| Condition:Freq | 0.001537 | 0.000587 | 2.619 | 0.008934 |

Observations: 1,177. Var(Item): <0.0001. Var(Subject): 0.0027.

##### M350 ~ Shared Template + Target Frequency

χ^2^(1) = 3.2751, *p* = 0.07034 when adding Condition to Frequency.

| Predictor | Estimate | SE | t-value | p-value (χ^2^) |
| --- | --- | --- | --- | --- |
| (Intercept) | 1.5933621 | 0.0911817 | 17.475 |  |
| Target Frequency | 0.0011876 | 0.0028698 | 0.414 | 0.001543 |
| Condition | 0.0221803 | 0.0156621 | 1.416 | 0.07034 |
| Condition:Freq | 0.0003720 | 0.0006028 | 0.617 | 0.5381 |

Observations: 1,177. Var(Item): <0.0001. Var(Subject): 0.0565.

##### M170 ~ Shared Root

| Predictor | Estimate | SE | t-value | p-value (χ^2^) |
| --- | --- | --- | --- | --- |
| (Intercept) | 1.655 | 0.05100 | 32.45 |  |
| Target Frequency | -0.00009232 | 0.0009182 | 0.10 | 0.9258 |
| Condition | 0.01634 | 0.01082 | 1.51 | 0.1316 |

Observations: 1,154. Var(Item): <0.0001. Var(Subject): 0.0209.

##### M350 ~ Shared Root

| Predictor | Estimate | SE | t-value | p-value (χ^2^) |
| --- | --- | --- | --- | --- |
| (Intercept) | 1.6512774 | 0.0581729 | 28.386 |  |
| Target Frequency | -0.0007466 | 0.0010404 | 0.718 | 0.4771 |
| Condition | 0.0192114 | 0.0122637 | 1.567 | 0.1186 |

Observations: 1,154. Var(Item): <0.0001. Var(Subject): 0.0028.

##### M170 ~ Shared Argument Structure

| Predictor | Estimate | SE | t-value | p-value (χ^2^) |
| --- | --- | --- | --- | --- |
| (Intercept) | 1.8142484 | 0.2196211 | 8.261 |  |
| Target Frequency | 0.0010719 | 0.0009813 | 1.092 | 0.2758 |
| Condition | -0.0186124 | 0.0480148 | 0.388 | 0.698 |

Observations: 1,143. Var(Item): <0.0001. Var(Subject): 0.0174.

##### M350 ~ Shared Argument Structure

| Predictor | Estimate | SE | t-value | p-value (χ^2^) |
| --- | --- | --- | --- | --- |
| (Intercept) | 1.8178339 | 0.2870062 | 6.334 |  |
| Target Frequency | 0.0006747 | 0.0006747 | 0.529 | 0.598 |
| Condition | -0.0168301 | 0.0624558 | 0.269 | 0.79 |

Observations: 1,143. Var(Item): <0.0001. Var(Subject): 0.0455.

# Rejection rates

## Experiment 1

| Condition | Rejection rate | Kept / Total |
| --- | --- | --- |
| +T +AS | 33.3% | 588 / 882 |
| -T -Rt | 34.1% | 581 / 882 |
| -T +Rt | 33.7% | 585 / 882 |
| Total | 33.7% | 1,754 / 2,646 |

There was no difference between conditions, χ^2^(2) = 0.042, p = 0.979.

## Experiment 2

| Condition | Rejection rate | Kept / Total |
| --- | --- | --- |
| -T +Rt | 34.2% | 580 / 882 |
| +T +AS | 32.7% | 594 / 882 |
| +T -AS | 34.7% | 576 / 882 |
| -T +AS | 35.5% | 569 / 882 |
| -T -Rt -AS | 34.9% | 574 / 882 |
| -V | 31.9% | 601 / 882 |
| Total | 34.0% | 3,494 / 5,292 |

There was no difference between conditions, χ^2^(5) = 1.335, p = 0.931.
